# Matters of the heart: cardiac toxicity of adjuvant systemic therapy for early-stage breast cancer

**DOI:** 10.3747/co.2008.173

**Published:** 2008-01

**Authors:** K. Towns, P.L. Bedard, S. Verma

**Affiliations:** * University of Toronto, Toronto, ON; † Sunnybrook Odette Cancer Centre, Toronto, ON

**Keywords:** Anthracyclines, trastuzumab, aromatase inhibitors, cardiac toxicity

## Abstract

Breast cancer remains the most common malignancy in women. Since the late 1980s, significant advances have been made in the treatment of this cancer. Those advances, particularly the ones in the adjuvant setting, have led to declines in the mortality associated with breast cancer. But another result has been treatments that are more complex and that potentially carry more toxicity. One key toxicity related to the adjuvant therapy of breast cancer is cardiac toxicity. Some of the agents commonly used for the treatment of breast cancer, including anthracyclines, trastuzumab, and possibly even aromatase inhibitors, have been associated with cardiac toxicity. The present article reviews the current understanding of cardiac toxicity risk and strategies to minimize cardiac morbidity associated with cytotoxic chemotherapy, trastuzumab therapy, and hormonal therapy with aromatase inhibitors for early-stage breast cancer.

## 1. INTRODUCTION

In Canada, breast cancer is the most common malignancy in women. During the last 15 years, an estimated 162,600 Canadian women were diagnosed with breast cancer, with more than 22,000 new cases diagnosed annually^1^. Following surgery, nearly all affected women will receive some form of adjuvant systemic therapy—cytotoxic chemotherapy, endocrine therapy, targeted therapy with a monoclonal antibody, or a combination—to reduce their risk of relapse and to improve survival. As a result of this improved adjuvant therapy and earlier detection with mammographic screening, the age-adjusted mortality of early-stage breast cancer continues to decline, with 96% and 86% of stage i and ii patients respectively expected to survive more than 5 years from initial diagnosis^1^.

Given a burgeoning population of long-term breast cancer survivors and an increasing recognition that breast cancer is a molecularly heterogeneous disease in which only subgroups of patients may benefit from specific adjuvant therapies^2^–^6^, minimizing the long-term side effects of treatment is now of utmost significance. Cardiac toxicity, which is among the most dreaded of the long-term complications of cancer treatment, has been reported with all three forms of adjuvant systemic therapy for breast cancer. The present article reviews the current understanding of cardiac toxicity risk and strategies to minimize cardiac morbidity associated with cytotoxic chemotherapy, trastuzumab therapy, and hormonal therapy with aromatase inhibitors (ais) for early-stage breast cancer.

## 2. ANTHRACYCLINE-INDUCED CARDIOTOXICITY

The anthracycline class of chemotherapeutic agents is commonly used to treat a variety of malignancies, including breast, gastric, and esophageal cancers, and sarcoma, lymphoma, and leukemia. A large meta-analysis of early clinical trials in breast cancer established the superiority of anthracycline-based adjuvant chemotherapy over the traditional cyclophosphamide, methotrexate, and 5-fluorouracil (cmf) regimen with an absolute 4% improvement in overall survival at 10 years^7^. Following the introduction of anthracyclines, more recent clinical trials have tried to define the optimal type, dose, and schedule of anthracycline administration; the value of adding agents such as taxanes and targeted therapies to an anthracycline backbone; and the possible alternatives to anthracycline-based therapy^8^,^9^.

Starting with their initial use in the 1960s, anthracyclines were recognized to potentially cause a variety of cardiac side effects. Rare acute toxicities include supraventricular tachycardia, electrocardiographic changes, ventricular ectopy, myopericarditis, and sudden death^10^. A serious long-term side effect of anthracycline therapy is the development of a dilated cardiomyopathy highlighted by systolic dysfunction and left-sided congestive heart failure^11^, which usually occurs 2–5 years after the last anthracycline dose. Late anthracycline-induced cardiomyopathy can develop insidiously, without significant evidence of systolic dysfunction on traditional measures of left ventricular ejection fraction (lvef)^12^ such as multiple gated acquisition scans or two-dimensional echocardiography. After the onset of symptoms of congestive heart failure (chf) secondary to anthracycline-induced cardiomyopathy, outcome is quite poor, with a median survival of approximately 1 year in a large population-based series^13^.

### 2.1 Pathogenesis

The pathophysiology of anthracycline-induced cardiomyopathy is poorly understood. Myocardial cells demonstrate a limited capacity to regenerate, and they are particularly susceptible to the degenerative effects of cytotoxic chemotherapy. Endomyocardial biopsies from patients with anthracycline-induced cardiomyopathy demonstrate irreversible myofibril loss, necrosis, and vacuolar degeneration^14^. Although numerous mechanisms have been proposed to account for these morphologic features, most studies suggest that anthracyclines cause myocardial damage through oxidative stress^15^. Anthracyclines form complexes with intracellular iron, leading to the generation of free radical species, lipid peroxidation, and depletion of antioxidants. These events leave myocardial cells susceptible to oxidative injury affecting nucleic acids, intracellular proteins, and mitochondria, which accumulate damage and ultimately trigger cell death.

### 2.2 Risk Factors for Anthracycline-Induced Cardiomyopathy

A number of risk factors are associated with the development of anthracycline-induced cardiomyopathy. A landmark retrospective review by Von Hoff *et al.*^16^ established a clear relationship between the cumulative dose of doxorubicin and the risk of chf. In their series, the incidence of chf after 400 mg/m^2^ of doxorubicin was 3%, rising to 7% at 550 mg/m^2^ and 18% at 700 mg/m^2^. Other traditional risk factors associated with anthracycline-induced cardiomyopathy include age greater than 70 years^16^,^17^, mediastinal radiation (concurrent or sequential)^17^–^21^, pre-existing cardiac disease^17^,^20^, hypertension^17^,^19^, and liver disease^22^. For reasons that are poorly understood, women are more susceptible to anthracycline-induced cardiomyopathy: as compared with men, they have a lower cumulative-dose threshold^23^.

### 2.3 Incidence of Anthracycline-Induced Cardiomyopathy

Because most multi-agent adjuvant breast cancer chemotherapy regimens deliver a cumulative dose of doxorubicin below 450 mg/m^2^, the risk of cardiac toxicity with anthracycline-based therapy was initially felt to be low. The early adjuvant trials of anthracyclines in breast cancer supported this notion of cardiac safety. In a meta-analysis by the Early Breast Cancer Trialists’ Collaborative Group, the mortality from heart disease was 0.08% annually in the anthracycline-treated group as compared with 0.06% annually in the non-anthracycline cmf group^7^. A retrospective analysis from a single centre reported a 1% incidence of chf at a median follow-up of 14 years in a group treated with doxorubicin–cmf; no chf events were reported in the cmf-only group^24^. However, these early reports included only relatively young patients with few medical comorbidities; they suffered from incomplete reporting and lacked prospective serial cardiac assessment.

More recent population-based studies suggest that the cardiac sequelae from anthracycline-based treatment may be much higher than previously thought. In a review of 31,748 women over the age of 65 years diagnosed with early-stage breast cancer in the Surveillance Epidemiology and End Results (seer)–Medicare database from 1992 to 1999, women who received doxorubicin-based adjuvant chemotherapy were 2.5 times more likely to be diagnosed with cardiomyopathy than were women who did not receive chemotherapy^25^. This increased risk of cardiomyopathy in the doxorubicin-treated group was observed despite a lower prevalence of pre-existing heart disease in the doxorubicin group before initiation of therapy. Similarly, a separate analysis of the seer database reported a 38.4% rate of chf at 10 years in anthracycline-treated women aged 66–70 years as compared with rates of 32.5% and 28% in women of the same age group who received non-anthracycline chemotherapy and no adjuvant chemotherapy respectively^26^,^27^.

Beyond clinically overt chf, anthracycline-based adjuvant therapy is also associated with subclinical cardiomyopathy. Longer follow-up of early adjuvant anthracycline-based clinical trials has demonstrated that administration of “safe” cumulative doses of doxorubicin below 450 mg/m^2^ may predispose to asymptomatic systolic dysfunction, regarded as a reduction of 10% or more from baseline in lvef. In the National Cancer Institute of Canada ma.5 trial, which randomized women with node-positive breast to 6 cycles of a cyclophosphamide, epirubicin, and 5-fluorouracil (cef) regimen or to 6 cycles of cmf, 25% of women who received cef developed an asymptomatic decline of 10% or more in lvef after 5 years; in the cmf group, just 9% developed an equivalent decline^28^. Similarly, the North Central Cancer Treatment Group (ncctg) N9831 Intergroup Adjuvant Trial, which used doxorubicin and cyclophosphamide (ac) followed by paclitaxel (t) as a backbone for the addition of trastuzumab therapy in women with human epidermal growth factor receptor type-2 (her2) overexpression, reported that 23.4% of patients experienced a 10% or greater decline in lvef following 4 cycles of ac^29^. The long-term clinical impact of this form of subclinical cardiomyopathy is unclear. However, an asymptomatic decline in lvef may limit the use of further systemic therapies that are potentially cardiotoxic. For example, in the N9831 trial, 7.5% of women would not have been eligible to receive trastuzumab with paclitaxel following 4 cycles of ac because of a decline in lvef from baseline^30^.

### 2.4 Strategies to Reduce Anthracycline-Induced Cardiomyopathy

The success of trastuzumab has rekindled enthusiasm for developing strategies to minimize anthracycline-induced cardiac dysfunction (Table I). Substituting epirubicin for doxorubicin reduces the incidence of clinical heart failure in the metastatic setting^31^. Prolonged infusion time and weekly administration of doxorubicin also cause less cardiotoxicity than the standard every-three-weeks bolus administration that is routinely used in clinical practice^32^, but the frequency of hospital visits for weekly administration and the shortage of resources for prolonged infusions make these options unappealing for patients and providers alike. Liposomal formulations of anthracyclines and the use of adjunctive scavengers of free radicals such as dexrazoxane have also been shown to reduce the incidence of chf in the treatment of metastatic disease^33^–^35^. However, no prospective clinical trials are currently evaluating these agents in the adjuvant setting. Beta-blockers^36^ and angiotensin converting-enzyme inhibitors^37^ used in combination with anthracyclines have also demonstrated promise; however, the single-centre studies of these combinations have involved small numbers of patients receiving a variety of chemotherapy combinations with limited long-term follow-up.

To be able to determine which patients should be targeted with cardioprotective strategies in future clinical trials, predictive markers of anthracycline-induced cardiac injury are needed. In a pooled analysis of three trials with serial monitoring of left ventricular function, more than one third of patients who went on to develop chf secondary to doxorubicin did not demonstrate a greater than 30% reduction in lvef before the onset of symptoms^12^. Monitoring of lvef is clearly an imprecise surveillance tool; it cannot reliably predict which patients with asymptomatic lvef decline are at risk of future cardiac events. Moreover, significant chf can occur without a preceding decline in lvef.

There is hope that alternative techniques of myocardial imaging may provide better discriminative power. In many other forms of cardiomyopathy, systolic dysfunction precedes a diastolic dysfunction. Indices of early diastolic dysfunction detectable by angiocardiography may predict anthracycline-induced cardiotoxicity more reliably than traditional multiple gated acquisition monitoring does^38^. Biochemical monitoring with troponin^39^–^43^ or B-type natriuretic peptide^44^ as early markers of anthracycline-induced myocardial injury may also eventually play a role.

Perhaps the most effective strategy for reducing the risk of cardiotoxicity is to limit anthracycline administration to the patients most likely to benefit from adjuvant anthracycline therapy. A recent U.S. Oncology Group trial demonstrated improved disease-free survival (dfs) for early-stage breast cancer treated with docetaxel and cyclophosphamide (tc) over standard ac^45^. As a result, it may be appropriate to use tc for early-stage breast cancer patients with a lower risk of cancer recurrence and risk factors for anthracycline cardiotoxicity. For patients with an elevated risk of breast cancer recurrence, in whom clinicians would be inclined to use an anthracycline followed by a taxane, it may be possible to use molecular markers such as co-amplification of topoisomerase iiα (*TOP2A*) and her2 as predictors of benefit from adjuvant anthracycline-based therapy^46^–^50^. However, the hypothesis that co-amplification of *TOP2A* and her2 predicts anthracycline sensitivity has yet to be prospectively validated in a clinical trial.

In the future, technology base on gene expression may provide valuable insights into which molecular subsets of breast cancer benefit from adjuvant chemotherapy and therefore warrant the risk of long-term toxicity. However, until prospective clinical trials establish effective alternatives to anthracyclines for patients with an elevated recurrence risk, more research is needed to define risk factors, predictive markers, and effective preventive strategies to minimize the burden of anthracycline-induced cardiotoxicity.

## 3. TRASTUZUMAB

Approximately 20% of breast cancers amplify or overexpress her2^51^ (sometimes both). Amplification or overexpression of her2 is associated with an aggressive breast cancer phenotype^52^. Tumours with her2 amplification or overexpression are more likely to demonstrate poor differentiation, high nuclear grade, and high proliferative rates. They are also associated with an increased risk of lymph-node metastasis and decreased estrogen and progesterone receptor expression^53^. These observations led to the development of trastuzumab (Herceptin: Genentech, San Francisco, CA, U.S.A.), a humanized monoclonal antibody against her2.

In 2001, a pivotal clinical trial by Slamon *et al.*^54^, which involved 469 patients with previously untreated her2-positive metastatic breast cancer, randomized patients to chemotherapy alone versus chemotherapy with trastuzumab. As compared with the chemotherapy-alone group, the chemotherapy-plus-trastuzumab group experienced increased time to disease progression (7.4 months vs. 4.6 months, *p* < 0.001) and an increased response rate (50% vs. 32%; *p* < 0.001)^54^. Similar results were seen when trastuzumab was studied in combination with docetaxel^55^. These encouraging results prompted study of trastuzumab in the adjuvant setting.

To date, five phase iii trials have studied trastuzumab in early-stage breast cancer: the ncctg Intergroup trial N9831, the National Surgical Adjuvant Breast and Bowel Project (nsabp) trial B-31 (which led to a combined analysis with ncctg N9831), the Herceptin Adjuvant (hera) trial, the Breast Cancer International Research Group (bcirg) trial 006, and the Finland Herceptin (Finher) trial.

### 3.1 Adjuvant Trastuzumab Trials for Early-Stage Breast Cancer

The ncctg N9831, hera, bcirg 006, and Finher trials evaluated the addition of trastuzumab to adjuvant chemotherapy for node-positive or high-risk node-negative her2-positive breast cancer; nsabp B-31 enrolled only node-positive patients (Table II)^56^–^60^. The most notable design differences across these trials involved sequential versus concurrent use of trastuzumab with adjuvant chemotherapy, and the administration of anthracyclines.

The nsabp B-31 trial evaluated concurrent use of trastuzumab with adjuvant chemotherapy. The ncctg N9831 trial involved two groups with regimens similar to those in the nsabp B-31 trial, but it also involved a third group that received sequential trastuzumab following adjuvant chemotherapy. This last group was not included in the joint analysis of these two trials, but its preliminary results provided insight on the relative efficacy of trastuzumab when used concurrently with, or sequentially after, the administration of adjuvant chemotherapy.

The hera trial provided data applicable to a wide range of chemotherapeutic regimens. In that study, 94% of participants received anthracyclines, and 26% received both anthracyclines and taxanes before randomization^56^. The hera trial will also provide data on duration of trastuzumab and whether 2 years of treatment with trastuzumab is more beneficial than 1 year of treatment.

The bcirg 006 trial had a third arm (a combination of docetaxel, carboplatin, and trastuzumab) that did not contain an anthracycline, providing information on whether trastuzumab is effective with non-anthracycline-based chemotherapy.

The Finher trial randomly assigned 1010 patients to docetaxel or vinorelbine followed by 5-fluorouracil, epirubicin, and cyclophosphamide for 3 cycles. The 232 her2-positive patients from this study were further randomized to trastuzumab or observation, and trastuzumab was given concurrently over 9 weeks with either docetaxel or vinorelbine.

The primary endpoint of all of the foregoing studies was either dfs or recurrence-free survival^56^–^60^. The secondary endpoints included overall survival and time to distant recurrence^56^–^60^. All of these trials have now reported an approximate 50% reduction in the risk of recurrence for patients assigned to receive adjuvant trastuzumab (Table II) 56–60.

### 3.2 Trastuzumab-Induced Cardiotoxicity

Early clinical trials with trastuzumab in patients with metastatic disease did not prospectively monitor for cardiac toxicity^51^,^62^. However, the independent Cardiac Review and Evaluation Committee (crec) retrospectively reviewed patients enrolled in seven phase ii and iii trials to evaluate the risk of cardiac dysfunction associated with trastuzumab^63^. The ac combination with trastuzumab had the highest rates of cardiotoxicity with a 27% incidence of cardiac dysfunction, including a 16% incidence of New York Heart Association (nyha) class iii/iv heart failure^63^. In comparison, cardiac dysfunction developed in 13% of patients treated with paclitaxel and trastuzumab (including 2% nyha class iii and iv chf) and in 3%–7% of patients treated with trastuzumab alone^63^. The crec concluded that, given the 25% improvement in overall survival associated with the use of trastuzumab in metastatic disease^55^, the benefits of trastuzumab in patients with her2-positive breast cancer outweighed the risk of cardiac dysfunction^63^. Also, based on the high rates of cardiac dysfunction noted in relevant populations, anthracyclines and trastuzumab should no longer be used concurrently in clinical practice. The observations by the crec led to the development of strict inclusion and exclusion criteria and guidelines for prospective cardiac monitoring in adjuvant trials.

#### 3.2.1 Cardiac Eligibility Criteria and Cardiac Monitoring

Most of the adjuvant trastuzumab trials had cardiac eligibility criteria that required a lvef of more than 50% and that excluded patients with a history of cardiac disease or cardiac risk factors (Table III)^56^,^58^,^59^,^61^,^64^. The hera trial was the exception, using a lvef cut-off of more than 55%. In the combined analysis of nsabp B-31 and ncctg N9831, patients were excluded from trastuzumab initiation if their lvef after anthracycline therapy was below the lower limit of normal or had declined by more than 15% from baseline, or if clinically significant cardiac symptoms had developed after treatment with anthracyclines^64^. Based on those criteria, 233 of 3497 patients (6.7%) who had completed doxorubicin and cyclophosphamide therapy were not able to initiate trastuzumab therapy^64^. Similarly, in the bcirg trial, approximately 2.4% of patients did not go on to receive trastuzumab after anthracycline therapy^61^.

The adjuvant trials also rigorously monitored lvef at baseline and throughout the study period, including after anthracycline administration. The hera trial has the longest prospective lvef follow-up of the adjuvant trials to date, with a final lvef assessment at 60 months after randomization^56^.

#### 3.2.2 Definition of Cardiac Toxicity and Criteria to Discontinue Trastuzumab

Although all five adjuvant trials evaluated cardiac dysfunction, their definitions of cardiac events and their guidelines for discontinuation or reintroduction of trastuzumab after documentation of a cardiac event showed subtle differences (Table III) 56,58,59,61,64. All five trials defined cardiac death and symptomatic heart failure as cardiac endpoints. However, they used variable definitions for significant lvef declines from baseline and absolute lvef cut-offs (Table III)^56^,^58^,^59^,^61^,^64^. The nsabp B-31 and ncctg N9831 trials defined cardiac endpoints as cardiac death or symptomatic heart failure (nyha iii/iv), confirmed by a cardiac review panel^30^; on the other hand, the hera trial used cardiac death or symptomatic heart failure (nyha iii/iv) with a drop in lvef of at least 10% from baseline and below an absolute value of 50%^56^. These differences across the trials make direct comparison of their results difficult. The hera, bcirg, and Finher trials also included asymptomatic declines in lvef as a cardiac endpoint, again using different lvef cut-offs in their definitions. The bcirg trial differed in that it also included grade 3 or 4 ischemia or infarction and grade 3 or 4 arrhythmia as cardiac endpoints^61^. The Finher trial also reported myocardial infarction as a cardiac endpoint^59^.

In the combined analysis and in the hera trial, the criteria for withholding or discontinuing trastuzumab are similar; however, hera uses a lower lvef cut-off of less than an absolute value of 45% (rather than 50%, as in the combined analysis). In nsabp B-31, ncctg N9831, hera, and bcirg 006, an absolute difference of more than 4% in the incidence of cardiac death or severe chf between the trastuzumab group and the observation group would have provoked early discontinuation of the study^62^. This circumstance did not occur in any of the trials.

### 3.3 Cardiac Risk in Trastuzumab Adjuvant Trials

In the trastuzumab adjuvant trials, the use of adjuvant trastuzumab was associated with an absolute increase in the risk of symptomatic chf of between 0.6% and 3.8% (Table IV)^30^,^57^–^60^,^66^. In the combined analysis of the nsabp B-31 and ncctg N9831 trials, 14.2% of patients (164 of 1159) had asymptomatic reductions in lvef before the planned completion of 52 weeks of trastuzumab therapy^65^. In the nsabp B-31 trial, 133 of 714 patients (19%) in the trastuzumab arm had to discontinue trastuzumab for symptomatic (31 patients) or asymptomatic (102 patients) declines in lvef 30.

In the hera trial, 72 participants (4.3%) discontinued trastuzumab because of cardiac problems^58^. As compared with the nsabp B-31 and ncctg N9831 trials, the hera trial also reported a lower incidence of severe chf in the trastuzumab group (0.6% vs. 3.8% and 3.5% respectively)^58^. It has been hypothesized that these differences can be attributed to the sequential rather than concurrent use of trastuzumab with chemotherapy, the longer time interval between completion of chemotherapy and initiation of trastuzumab administration (3 months on average in the hera trial), and the higher lvef required as inclusion criteria in the hera trial (above 55% as compared with above 50% in the combined analysis)^58^.

The bcirg 006 trial reported a significant difference in symptomatic cardiac events between the ac/docetaxel group and the ac/docetaxel/trastuzumab group^61^. However, no statistically significant difference in cardiac events was observed between the ac/docetaxel arm and the docetaxel/carboplatin/trastuzumab arm^61^. Also, at 23 months’ follow-up, no statistically significant difference in dfs was observed between the ac/docetaxel/trastuzumab (anthracycline-containing) arm and the docetaxel/carboplatin/trastuzumab (non-anthracycline-containing) arm (hazard ratios of 0.49 and 0.61 respectively)^61^, suggesting that non-anthracycline/trastuzumab–containing regimens may be as effective as anthracycline/trastuzumab–containing regimens, with a lower rate of cardiac toxicity.

The only trial that reported no increased risk of cardiac toxicity in the trastuzumab group was Finher. Surprisingly, an increased risk of symptomatic and asymptomatic heart failure was observed in the non-trastuzumab arm (statistical significance not reported)^59^. The major differences in this trial were the smaller study population (*n* = 232) and the duration of trastuzumab therapy (9 weeks).

#### 3.3.1 Risk Factors for Trastuzumab-Induced Cardiotoxicity

The risk factors for trastuzumab-induced cardiotoxicity have not been clearly defined. The nsabp B-31 trial reported increased age (*p* = 0.03), baseline lvef of 50%–54% (*p* = 0.0003), and post-anthracycline lvef of 50%–54% (*p* < 0.0001) as significant risk factors^30^. Interestingly, this study also showed a suggestion of increased risk with the use of antihypertensive medications before study entry (*p* = 0.02)^30^. Of note, left-sided radiation, cardiac arrhythmias, and other cardiac risk factors were not found to be risk factors for cardiac toxicity in nsabp B-31 or ncctg N98312^30^,^66^. In the hera trial, a higher cumulative dose of doxorubicin (287 mg/m^2^ vs. 257 mg/m^2^) or epirubicin (480 mg/m^2^ vs. 422 mg/m^2^), a lower screening lvef, and a higher body mass index were all associated with trastuzumab-associated cardiac dysfunction^67^. The other trials have not yet reported analyses of the predictive factors of trastuzumab-induced cardiac dysfunction.

#### 3.3.2 Pathogenesis

Little is known about the mechanism of trastuzumab-induced cardiac dysfunction. The transmembrane tyrosine kinase receptor her2 (ErbB2) promotes cell proliferation when activated^51^. To study the role of ErbB2 signalling in cardiac tissue, mice with a cardiac-restricted deletion of *ERBB2* were bred^68^,^69^. The mice were viable and displayed no overt deficits at birth. Over time, the ErbB2-deficient mice were found to develop features of dilated cardiomyopathy, including chamber dilation, wall thinning, and decreased contractility^68^,^69^. They were also found to be more susceptible to anthracycline-induced cardiac dysfunction^68^. As a result, the authors suggested that ErbB2 signalling might have a role in myocytes in the prevention of dilated cardiomyopathy triggered by environmental stressors^68^,^69^.

In contrast to anthracycline cardiac toxicity, which is irreversible, dose-dependent, and associated with ultrastructural changes (irreversible myofibril loss, necrosis, and vacuolar degeneration)^14^, trastuzumab-associated cardiac toxicity is thought to be reversible, idiosyncratic, and not associated with structural damage. In a series investigated by the M.D. Anderson Cancer Center, 9 of 38 patients with cardiac toxicity underwent right ventricular endomyocardial biopsies; no ultrastructural changes were seen^70^. This lack of ultrastructural damage may explain the reversible nature of trastuzumab-related cardiotoxicity as observed in clinical studies (Perez EA, Suman VJ, Davidson N, *et al.* Advances in monoclonal therapy for breast cancer: further analysis of ncctg N9831. Presented at the 41st Annual Meeting of the American Society of Clinical Oncology; May 16, 2005; Orlando, FL).

#### 3.3.3 Reversibility of Trastuzumab-Induced Heart Failure

A retrospective review of 38 patients with trastuzumab-induced cardiotoxicity at M.D. Anderson revealed that the cardiotoxicity might be reversible^70^. Trastuzumab was discontinued in 37 of these patients, and 31 received standard heart failure treatment, which included both beta-blockers and angiotensin converting-enzyme inhibitors^70^. All 37 patients showed improvement in lvef at 1–3 months. Two patients had persistent left ventricular dysfunction at 6 months^70^. Of the 37 patients, 25 (66%) were rechallenged with trastuzumab (once stability or improvement of left ventricular dysfunction occurred), and only 3 (12%) developed recurrent left ventricular dysfunction or symptoms of chf (or both) prompting discontinuation of further trastuzumab^70^. These results provided the first suggestion that it may be safe to reintroduce trastuzumab in patients who develop cardiac dysfunction, once their symptoms or ejection fraction improve with medical therapy.

In the nsabp B-31 trial, of the 31 patients in the trastuzumab group who met criteria for symptomatic chf 6 months after the onset of heart failure, 26 were asymptomatic, 1 demonstrated ongoing symptoms of chf, and 18 remained on cardiac medications^64^. At 6 months after discontinuation of trastuzumab for either symptomatic chf or asymptomatic decline in lvef, 25% of patients continued to show a lvef below 50%^30^. Data from ncctg N9831 and bcirg 006 are also consistent with this persistent decline in lvef after discontinuation of trastuzumab. Of the 14 patients who had a cardiac event in the ncctg N9831 trial, 29% continued to show a lvef below 50%^67^. In the bcirg 006 trial, 180 of 1040 participants (17.3%) treated with ac, paclitaxel, and trastuzumab had a more than 10% reduction in lvef relative to baseline. At 42 days, 26% of the participants (37 of 145) had a persistent decline in lvef on repeat measurement 61. These results led to the recognition that, although most patients recover contractility, a significant proportion of patients (approximately 25% in the trials already mentioned) experience persistent contractile dysfunction. Moreover, the long-term implications of trastuzumab-associated lvef decline—even in patients who subsequently recover to baseline lvef—are unknown.

### 3.4 Weighing the Risks and Benefits of Trastuzumab

Ultimately, the risks and benefits of using trastuzumab must be weighed for patients on an individual basis, taking into account the exclusion criteria used in the adjuvant trials and the risk factors mentioned earlier. Many questions remain unanswered: sequential versus concurrent use of trastuzumab, the necessity of anthracyclines, the long-term significance of asymptomatic declines in lvef, and the exact pathogenesis and risk factors associated with trastuzumab-induced cardiotoxicity. Also, to date, cardiac toxicity has been evaluated based on systolic function. Little is known about possible diastolic dysfunction associated with trastuzumab.

The ncctg N9831 trial found a slightly decreased incidence of chf with sequential rather than concurrent use of trastuzumab with chemotherapy (2.5% vs. 3.5%)^62^. However, initial reports have suggested that, as compared with concurrent administration, sequential administration of trastuzumab may be less efficacious at preventing breast cancer recurrence (Perez EA, Suman VJ, Davidson N, *et al.* Advances in monoclonal therapy for breast cancer: further analysis of ncctg N9831. Presented at the 41st Annual Meeting of the American Society of Clinical Oncology; May 16, 2005; Orlando, FL). Nevertheless, the fundamental message of the five trials is unmistakably clear: trastuzumab, regardless of strategy of administration, clearly has an important role in reducing the risk of recurrence of her2-positive breast cancer. The challenge for the next generation of clinical studies is therefore to find the means of delivering trastuzumab in the most efficacious and least cardiotoxic manner.

Currently, the challenge for clinicians deciding between sequential and concurrent treatment is to balance their patients’ cardiac risk with the risk of tumour recurrence and to discuss this balance with their patients—especially when the likelihood of benefit may be low for some patients. With this in mind, the intriguing results of bcirg 006 raise the question of whether anthracyclines, with their attendant risk of cardiac dysfunction, can be omitted in the management of her2-positive breast cancer, particularly for patients at high risk of trastuzumab cardiotoxicity^61^. This question is significant, given that 6.7% of patients who completed doxorubicin and cyclophosphamide therapy in nsabp B-31 and ncctg N9831 were not able to initiate trastuzumab therapy because of a decline in lvef after receiving anthracyclines^64^. The question of the clinical significance and long-term outcome of asymptomatic decline in lvef also remains. Longer follow-up in all of the adjuvant trials is needed to assess the implications of the asymptomatic lvef declines seen in nsabp B-31, ncctg N9831, hera, and bcirg 006. Until then, the balancing act will continue.

## 4. AROMATASE INHIBITORS

Aromatase inhibitors are used in postmenopausal women with hormone receptor–positive breast cancer. They act by inhibiting aromatase, the enzyme responsible for converting androgens to estrogens, thereby reducing estrogen levels. Unlike tamoxifen, ais are not partial estrogen agonists. Tamoxifen acts as a selective estrogen receptor modulator at the estrogen receptor. Because tamoxifen is a partial estrogen agonist, it is associated with an increased risk of thromboembolic events and uterine cancer, and it protects against bone demineralization. Aromatase inhibitors are not used alone in premenopausal women, because they cause a reduction in negative feedback on the hypothalamic–pituitary axis and thereby stimulate gonadotropin secretion, which stimulates the ovaries to produce more androgens, ultimately increasing estrogen levels^71^.

As compared with tamoxifen, third-generation ais have been shown to improve dfs in early breast cancer. They can be given as first-line hormonal therapy^72^,^73^, after 2–3 years of treatment with tamoxifen (switching to an ai rather than completing 5 years with tamoxifen)^74^–^76^, or as extended treatment following completion of 5 years of tamoxifen^77^. The switch strategy has also been associated with an over-all survival benefit in her2-positive patients^75^.

### 4.1 AIs and Cardiac Toxicity

#### 4.1.1 First-line Hormonal Therapy Trials

The Breast International Group (big) 1–98 trial is a four-arm trial of letrozole as compared with tamoxifen for 5 years. The two additional arms were established to evaluate the early-switch strategy to tamoxifen from letrozole or to letrozole from tamoxifen. So far, only the results of upfront letrozole alone as compared with tamoxifen alone have been reported.

Patients on letrozole experienced improved dfs, but overall survival did not differ significantly between groups (Table V)^74^,^76^–^80^. The rate of all adverse cardiac events was 4.8% in the letrozole group and 4.7% in the tamoxifen group (*p* = 0.87)^78^. Concerns arose when it was noted that, as compared with women in the tamoxifen arm, women in the letrozole arm had significantly more grades 3, 4, and 5 cardiac events (based on the Common Toxicity Criteria, version 2, of the U.S. National Cancer Institute: 2.4% vs. 1.21%, *p* = 0.001)^78^. These data must be interpreted with caution because baseline cardiovascular disease, lvef, and cardiac risk factors were not reported. The letrozole group also experienced a lesser improvement in cholesterol profile than did the tamoxifen group^78^. The Arimidex, Tamoxifen Alone or in Combination trial also found a statistically significant improvement in dfs with anastrozole (Arimidex: AstraZeneca Pharmaceuticals, Wilmington, DE, U.S.A.) as compared with tamoxifen^79^. That study detected no significant difference in the rates of ischemic cardiovascular disease between groups^79^.

#### 4.1.2 Switching to an AI After 2–3 Years of Tamoxifen Versus 5 Years of Tamoxifen

The Intergroup Exemestane Study (ies), a combined analysis of the Austrian Breast and Colorectal Cancer Study Group (abcsg) 8 study and the Arimidex–Nolvadex (arno)–95 trial, and the Italian Tamoxifen Anastrozole (ita) trial all compared tamoxifen alone for 5 years with 2–3 years of tamoxifen followed by an ai to reach a full 5 years of hormonal therapy. The switch to an ai was associated with a significantly longer dfs in all of those trials^74^,^76^,^80^. In the ies trial, a trend towards increased cardiac events with exemestane as compared with tamoxifen was noted^80^. None of the trials found a statistically significant difference in cardiac events between the ai and tamoxifen groups^74^,^76^. In the ita trial, a greater rate of lipid metabolism disorders (not defined in the publication from that study) was seen in the anastrozole arm (8.1% vs. 1.4% in the tamoxifen group, *p* = 0.01). No comparison to baseline lipid profiles was reported. Notably, the ies trial and the combined analysis of abcsg-8 and arno-95 excluded patients with a “clinically significant cardiac disorder” or “uncompensated cardiac insufficiency”^74^,^75^. However, without clear reporting of baseline cardiovascular risk factors, it is difficult to know how to apply the results of these safety analyses to patients with an elevated risk of cardiovascular disease.

#### 4.1.3 Extended Treatment with AIs After Completion of 5 Years of Tamoxifen

The ma.17 trial reported no significant difference in its ai and placebo arms with regard to the rate of cardiovascular events after 5 years of tamoxifen^77^. The rates of hypercholesterolemia were 11.9% in the letrozole group and 11.5% in the placebo group (*p* = 0.67)^77^. This trial reported previous diagnoses of cardiovascular disease at baseline, and rates of cardiovascular disease were comparable in both groups (letrozole: 12%; placebo: 11%). Notably, the ma.17 trial is the only trial that compared an ai to placebo rather than to tamoxifen, providing a clearer assessment of the cardiac safety of ais. However, given that all of the patients had previously been treated with tamoxifen for 5 years, it is difficult to determine the protective effect that that treatment may or may not have had, and how to interpret the data for patients who will be treated with 5 years of ai therapy up front, without tamoxifen.

### 4.2 Are AIs Cardiotoxic?

Based on the foregoing data, ais appear to be associated with a slightly greater incidence of cardiovascular endpoints; however, such comparisons are not statistically significant in most of the adjuvant ai trials. Nevertheless, it must be remembered that these trials were not designed to address the issue of cardiac disease. By in large, they did not clearly define criteria for cardiac endpoints or prospective evaluation of cardiac safety^81^. In addition, clinical follow-up from these trials is currently short, and longer follow-up is required to determine the true cardiac safety of ais in the long term.

## 5. SUMMARY

The advances made in adjuvant systemic therapy have been very successful in reducing the mortality rate associated with breast cancer. More women are now living with breast cancer and more patients are also now receiving a combination of therapies: chemotherapy, targeted therapy, and endocrine treatments. Therefore, addressing the long-term toxicity of treatments is critical.

The present article has highlighted cardiac toxicity as one of the key long-term toxicities of adjuvant systemic therapy. It is important for all practitioners to recognize this toxicity and to make an attempt to reduce its onset by selecting appropriate patients for adjuvant therapy, by selecting appropriate therapy based on patient factors and risk of recurrence, and by increasing awareness and education in patients and allied health care staff about cardiac toxicity. Although optimizing adjuvant therapy to reduce the risk of breast cancer recurrence is critical, recognizing and managing related toxicity is also important.

## Figures and Tables

**TABLE I:** Risk factors and strategies to prevent anthracycline-induced cardiotoxicity

Risk factors	Prevention strategies
Cumulative anthracycline dose	Alternative anthracyclines
Increased age	Liposomal formulations
Mediastinal radiation	Weekly administration
Pre-existing cardiac disease	Prolonged infusion
Hypertension	Adjunctive dexrazoxane
Liver disease	? Adjunctive beta-blockers or
Female sex	Angiotensin converting-enzyme inhibitors

**TABLE II:** Clinical trial design and efficacy results for trials of adjuvant trastuzumab in early-stage breast cancer

Trial	Regimen	Patients (*n*)	Median follow-up (months)	dfs (%)	hr (95% ci/*p* value)	Overall survival (%)	hr (95% ci/*p* value)
Combined analysis (nsabp B-31, ncctg N9831)^57^	ac→paclitaxel	1989	36	73.1	0.48 (0.41 to 0.57/<0.00001)	89.4	0.65 (0.51 to 0.84/0.0007)
	ac→paclitaxel plus trastuzumab for 1 year	1979		85.9		92.6	
ncctg N9831	ac→paclitaxel→trastuzumab	985					
hera^56^,^58^	Chemotherapy→observation	1698	23.5	74.3	0.64 (0.54 to 0.76/<0.0001)	89.7	0.66 (0.47 to 0.91/0.0115)
	Chemotherapy→trastuzumab for 1 year	1703		80.6		92.4	
	Chemotherapy→trastuzumab for 2 years	1701					
bcirg 006^60^,^61^	ac→docetaxel	1073	36	77		86	
	ac→docetaxel plus trastuzumab for 1 year	1074		83	0.61 (0.48 to 0.76/<0.0001 compared with ac→docetaxel group)	92	0.59 (0.42 to 0.85/0.004 compared with ac→docetaxel group)
	Docetaxel plus carboplatin plus trastuzumab for 1 year	1075		82	0.67 (0.54 to 0.83/0.0003 compared with ac→docetaxel group)	91	0.66 (0.47 to 0.93/<0.017 compared with ac→docetaxel group)
Finher^59^	Docetaxel→fec	58	35	77.6		89.7	
	Vinorelbine→fec	58					
	Docetaxel plus trastuzumab (9 weeks) →fec	54	37	89.3	0.42 (0.21 to 0.83/0.01)	96.3	0.41 (0.16 to 1.08/0.07)
	Vinorelbine plus trastuzumab (9 weeks) →fec	62 (her2-positive subset)					

dfs = disease-free survival; hr = hazard ratio; ci = confidence interval; nsabp = National Surgical Adjuvant Breast and Bowel Project; ncctg = North Central Cancer Treatment Group; ac = doxorubicin, cyclophosphamide; fec = fluorouracil, epirubicin, cyclophosphamide.

**TABLE III:** Cardiac criteria for trials of adjuvant trastuzumab in early-stage breast cancer

	nsabp B-31^30^,^64^	ncctg N9831^64^	hera^56^,^58^	bcirg 006^60^,^61^	Finher^59^
Cardiac exclusion criteria	lvef < lln History of angina pectoris requiring anti-angina medication arrhythmias requiring medication severe conduction abnormality clinically significant valvular disease cardiomegaly on chest radiograph poorly controlled hypertension mi, chf, or cardiomyopathy left ventricular hypertrophy on echocardiography	lvef < lln History of angina pectoris requiring anti-angina medication arrhythmias requiring medication severe conduction abnormality clinically significant valvular disease cardiomegaly on chest radiograph Poorly controlled hypertension mi, chf, or cardiomyopathy clinically significant pericardial effusion	lvef < 55% by echocardiography or muga after all chemotherapy and radiotherapy History of documented chf cad with previous Q-wave mi angina pectoris requiring medication uncontrolled hypertension clinically significant valvular disease unstable angina	Full description not yet published Approximately, age > 70 years lvef < 50% at baseline	Age > 66 years Severe hypertension Cardiac disease, including heart failure of any degree arrhythmia requiring regular medication mi in the last 12 months
Cardiac monitoring	Assessment of lvef with muga before randomization and at 3, 6, 9, 18 months after randomization	Assessment of lvef with muga or echocardiography before randomization and at 3, 6, 9, 18 months after randomization	Responses to a cardiac questionnaire, physical exam, 12-lead ecg, and assessment of lvef with muga or echocardiography at baseline, and at 3, 6, 12, 18, 24, 30, 36, 60 months after randomization	Similar to nsabp B-31	Assessment of lvef with muga or echocardiography at baseline, after the last fec cycle, and 12 and 36 months after chemotherapy
Cardiac endpoint definitions	Definitive or probable cardiac death Heart failure with nyha class iii/iv symptoms	Definitive or probable cardiac death Heart failure with nyha class iii/iv symptoms Asymptomatic (nyha class i) or mildly symptomatic (nyha class ii) with declines in lvef of 10% or more from baseline to a level of 50% or less	Cardiac death Severe chf defined as nyha class iii or iv (functional class confirmed by a cardiologist) **and** a decrease in lvef of at least 10% below baseline and to less than 50% Grade 3 or 4 ischemia/infarction Grade 3 or 4 arrhythmias Asymptomatic lvef decline, defined as an absolute lvef decline of more than 15% or lvef below the lower limit of normal	Definitive or probable cardiac death Symptomatic chf mi Asymptomatic decline in lvef of more than 15% from baseline or of more than 10% from baseline resulting in an lvef of less than 50%	Cardiac death Symptomatic chf
Criteria to hold trastuzumab	Asymptomatic decline in lvef of more than 15% or to below the lln	Asymptomatic decline in lvef of more than 15% or to below the lln	Asymptomatic, and lvef of 45% or lower or lvef of 50% or lower and declined by at least 10% from baseline	Similar to nsabp B-31	Not reported
Criteria to discontinue trastuzumab	Symptomatic cardiac dysfunction while receiving trastuzumab In asymptomatic patients, lvef fails to recover to above lln after trastuzumab held for 4 weeks	Symptomatic cardiac dysfunction while receiving trastuzumab In asymptomatic patients, lvef fails to recover to above lln after trastuzumab held for 4 weeks	Symptomatic heart failure **and** lvef < 45% or lvef < 50% and declined by at least 10% from baseline Asymptomatic patients whose lvef fails to recover after trastuzumab held for 3 weeks Patients in whom trastuzumab was re-introduced and who subsequently experienced another decline in lvef	Similar to nsabp B-31	Not reported

nsabp = National Surgical Adjuvant Breast and Bowel Project; ncctg = North Central Cancer Treatment Group; hera = Herceptin Adjuvant (Trial); bcirg = Breast Cancer International Research Group; Finher = Finland Herceptin (Trial); lvef = left ventricular ejection fraction; lln = lower limit of normal (assumed to be 50%); mi = myocardial infarction; chf = congestive heart failure; muga = multiple gated acquisition; cad = coronary artery disease; ecg = electrocardiograph; fec = fluorouracil, epirubicin, cyclophosphamide; nyha = New York Heart Association.

**TABLE IV:** Cardiac toxicity in the trials of adjuvant trastuzumab in early-stage breast cancer

Trial	Regimen	Patients (*n*)	Follow-up (months)	Cardiac death (%)	Severe chf— nyha iii/iv (%)	Symptomatic chf, including severe (%)	Decrease in lvef by 15% or more from baseline (%)	Discontinued trastuzumab because of cardiac problems (%)
nsabp B-31^57^,^64^	ac→paclitaxel	814	60 [36]	0.1 (*n*=1)	0.9	[1]	nr	na
	ac→paclitaxel plus trastuzumab for 1 year	850		0.0	3.8	[5.1]	[14]	[19]
ncctg N9831^57^,^66^	ac→paclitaxel		36	0.1 (*n*=1)	0.3	nr	6.7	na
	ac→paclitaxel plus trastuzumab for 1 year			0.0	3.5		17.3	15.4
	ac→paclitaxel→ trastuzumab				2.5			
hera^58^	Chemotherapy → observation	1708	24	0.06 (*n*=1)	0.00	0.12	2.1^a^	na
	Chemotherapy→ trastuzumab for 1 year	1678		0.00 *p*=1.0	0.60 *p<*0.0001	2.15 *p<*0.0001	7.0^a^	4.3 *p<*0.0001
bcirg 006^60^	ac→docetaxel	1073	36	0.0	0.4	na	10.1	na
	ac→docetaxel plus trastuzumab for 1 year	1074		0.0	1.9	18.1	nr	
	Docetaxel plus carboplatin plus trastuzumab for 1 year	1075		0.0	0.4	8.6	nr	
Finher^59^	Docetaxel→fec	58	35	0.0	2.8	na	6.0	na
	Vinorelbine→fec	58			(*n*=4)^b^			
	Docetaxel plus trastuzumab (9 weeks)→fec	54		0.0	0.0^b^		3.5	nr
	Vinorelbine plus trastuzumab (9 weeks)→fec	62	37					

a Decrease in left ventricular ejection fraction by 10% or more from baseline and below 50%.

b Includes infarction and cardiac failure.

chf = congestive heart failure; nyha = New York Heart Association class; lvef = left ventricular ejection fraction; nsabp = National Surgical Adjuvant Breast and Bowel Project; ac = doxorubicin, cyclophosphamide; nr = not reported; na = not applicable; ncctg = North Central Cancer Treatment Group; hera = Herceptin Adjuvant (Trial); bcirg = Breast Cancer International Research Group; Finher = Finland Herceptin (Trial); fec = fluorouracil, epirubicin, cyclophosphamide.

**TABLE V:** Summary of aromatase inhibitor trials: efficacy and cardiac events

Trial	Regimen	Median follow-up	Approximate hr for dfs/efs (95% ci/*p* value)	Cardiac events [% (*p* value)]
big-1.98^78^	Letrozole alone Tamoxifen alone	51 months	0.82 (0.71 to 0.95/0.007)	All cardiac events: 2.4 vs. 1.4 (0.001) Ischemic heart disease (overall, grades 3–5): 1.1 and 0.7 (0.06) Cardiac failure (overall, grades 3–5): 0.7 and 0.3 (0.04)
atac^79^	Anastrozole alone Tamoxifen alone	68 months	0.87 (0.78 to 0.97/0.01)	Ischemic cardiovascular disease: 4.1 vs. 3.6 (0.1)
ies^80^	Tamoxifen→exemestane Tamoxifen alone		0.74 (0.64 to 0.85/0.0001)	Myocardial infarction: 1.3 vs. 0.8 Angina: 7.1 vs. 6.5
Combined analysis: abcsg-8 and arno-95^74^	Tamoxifen→anastrozole Tamoxifen alone	28 months	0.61 (0.42–0.87/0.0009)	Myocardial infarction: <1% vs. <1% (1.0)
ita^76^	Tamoxifen→anastrozole Tamoxifen alone	64 months	0.57 (0.38 to 0.85/0.01)	Cardiovascular disease: 7.6 vs. 6.2 (0.6)
ma.17^77^	Tamoxifen for 5 years→letrozole Tamoxifen for 5 year→placebo	2.4 years	0.57 (0.43 to 0.75/0.00008)	Cardiovascular events: 4.1 vs. 3.6 (0.24)
